# Sterile Versus Non-Sterile Gloves in Dental Extractions: A Systematic Review and Meta-Analysis

**DOI:** 10.3390/cmtr19010006

**Published:** 2026-01-19

**Authors:** Mustafa Mohammad Ali Saffar, E. Krabbendam, E. B. Wolvius, J. T. van der Tas

**Affiliations:** 1 Department of Oral & Maxillofacial Surgery, Erasmus Medical Center, 3015 GD Rotterdam, The Netherlands; 2 Medical Library, Erasmus Medical Center, 3015 GD Rotterdam, The Netherlands

**Keywords:** dental infection control, protective gloves, oral surgical procedures, surgical wound infection, wound healing, postoperative complications, mouth microbiology

## Abstract

Healthcare-associated infections remain an ongoing concern across medical and dental practice, prompting continuous evaluation of infection prevention measures. In dental extractions, the necessity of sterile gloves is debated, as the oral cavity represents an inherently contaminated environment. This systematic review and meta-analysis evaluated whether the use of sterile gloves reduces postoperative socket infections compared with non-sterile gloves. A search of MEDLINE, Embase, Web of Science, Cochrane CENTRAL, and Google Scholar identified randomized controlled trials, clinically controlled trials, and observational trials directly comparing sterile versus non-sterile glove use during dental extractions. The primary outcome of this study was extraction socket infection at day 7 post-surgery. A meta-analysis using relative risk (RR) was performed for dichotomous data. Of the initial 7170 publications found, seven articles met inclusion criteria. Infection rates ranged from 0% to 3.9%, with an overall infection rate of 0.3% in the sterile glove group (672 patients) and 1.3% in the non-sterile glove group (758 patients). Three studies qualified for meta-analysis, resulting in an RR of 0.30 (95% CI 0.07–1.24), indicating no significant difference in postoperative infections between sterile and non-sterile glove usage. Given the limitations of small sample sizes, low event rates, incomplete reporting, and lack of subgroup data for surgical versus non-surgical extractions, no difference in postoperative infection was found between sterile and non-sterile glove use. Additional research is needed to determine whether glove sterility influences infection risk, particularly in surgical procedures.

## 1. Introduction

Effective infection-control policies are essential in dental practice due to routine exposure to saliva, blood, aerosols, and oral microorganisms, which present significant risks for cross-infection. Pathogen transmission can occur through direct contact with contaminated instruments, gloves, and oral tissues, while dental procedures also generate aerosols and splatter that facilitate microbial dissemination within the clinical environment, thus necessitating structured infection-control protocols [1]. Standard infection-control measures in dentistry include hand hygiene, use of personal protective equipment (PPE) such as gloves, masks, and eye protection, surface disinfection, and instrument sterilization. These interventions are designed to limit the transmission of pathogens between patients and clinicians and are supported by evidence demonstrating their effectiveness in reducing microbial contamination and infection risk [2,3,4].

The use of sterile gloves in dental extractions has been debated for several decades. Notably, Giglio et al. [5] conducted one of the initial comparative studies in 1993. Despite continued research and the passage of time, a consensus regarding the use of sterile gloves in surgical and non-surgical dental extractions remains absent.

The World Health Organization (WHO) patient safety workgroup recommends the use of sterile gloves during surgical procedures to minimize the dissemination of pathogens and protect both patients and healthcare workers from germ transmission [6]. This recommendation is re-iterated in the current Centers for Disease Control and Prevention (CDC) guidelines regarding the use of personal protective equipment in dental care [7]. These guidelines are of importance as they underscore the current standard in dental care and oral surgery. However, the oral cavity can be considered as an inherently contaminated surgical field. Previous research has demonstrated that the oral cavity harbors a diverse spectrum of over 700 bacterial species, including aerobic and anaerobic bacteria [8]. Since sterile gloves quickly become contaminated upon entering the mouth, there is questionable rationale for assuming sterile gloves provide microbiological advantage over non-sterile gloves in surgical extractions. Therefore, the efficacy of sterile gloves to reduce infection rates in surgical dental extractions has raised questions. Currently, expert opinions tend to shape infection prevention protocols regarding glove usage in surgical as well as non-surgical extractions.

The question on glove use in dental extractions is of importance from both a clinical and an environmental perspective. Healthcare systems as a whole are responsible for 4% to 5% of total emissions of greenhouse gas emissions (GHG) worldwide [9]. Therefore, there is growing need for measures to reduce greenhouse gas emissions and contribute to more sustainable healthcare. In this light, the use of sterile gloves in healthcare has been a topic of discussion because of their comparatively greater environmental impact. Compared to non-sterile gloves, sterile gloves cause an 11.6-fold higher waste production [10]. If current literature shows that the use of sterile gloves during dental extractions does not provide additional health benefits such as the prevention of infections, their routine may be considered unnecessary. This could ultimately lead to the abolishment of the use of sterile gloves in dental extractions. In addition, the literature on waste reduction in health care, can provide more awareness and support for further research to reduce greenhouse gas emissions. From a cost-effectiveness perspective, the possible abolishment of sterile gloves in dental extractions would reduce costs significantly. Sterile gloves are more expensive given their additional treatment and packaging, and dental extractions are one of the most common outpatient procedures performed.

In a prior systematic review and meta-analysis from 2016, no difference in infection rate was reported between the use of sterile and non-sterile gloves in outpatient surgical procedures, including dental extractions [11]. However, this study did not distinguish between surgical and non-surgical extractions. This distinction is relevant as sterile gloves are currently only recommended in surgical extractions and surgical extractions are associated with higher infection rates [12]. The aim of this systematic review is to provide an updated, comprehensive overview of potential differences in infection rates after dental extractions, comparing sterile and non-sterile gloves. Moreover, this review aims to perform a sub-analysis by categorizing surgical and non-surgical extractions to assess potential differences in infection rates associated with the use of sterile and non-sterile gloves.

To address these aims, our systematic review and meta-analysis was conducted to evaluate whether new evidence has emerged, especially between 2016 and 2025, given the absence of consensus on glove use in dental extractions.

## 2. Materials and Methods

### 2.1. Search Strategy

The search strategy was guided by the following research question: Does the use of sterile gloves, compared with non-sterile gloves, reduce postoperative infection rates following surgical or non-surgical dental extractions? Eligibility criteria were structured using a PICOT framework: studies were included if they involved patients undergoing dental extractions (Population), compared sterile gloves (Intervention) to non-sterile gloves (Comparator), and reported postoperative socket infection as the primary outcome at a minimum follow-up of 7 days (Outcome).

The methods in this review are described based on the Preferred reporting items for systematic reviews and meta-analyses (PRISMA) Checklist [13] and the Prisma-S extension to the PRISMA Statement for Reporting Literature Searches in Systematic Reviews [14]. An exhaustive search strategy was developed by an experienced information specialist (EK) in cooperation with the first author (MMA). The search was developed in Embase.com, optimized for sensitivity and then translated to other databases following the method as described by Bramer et al. [15]. The search was carried out in the databases Medline ALL via Ovid (1946 to Daily Update), Embase.com (1971–present), Web of Science Core Collection (Science Citation Index Expanded (1975–present); Social Sciences Citation Index (1975–present); Arts & Humanities Citation Index (1975–present); Conference Proceedings Citation Index-Science (1990–present); Conference Proceedings Citation Index-Social Science & Humanities (1990–present) and Emerging Sources Citation Index (2015–present)) and the Cochrane Central Register of Controlled Trials via Wiley (1992–present). Additionally, a search was performed in Google Scholar from which the 200 most relevant references were downloaded using the software Publish or Perish version 8 [16]. The search was last updated in July 2025, using the methods described by Bramer et al. [17].

The search strategies for Medline and Embase used relevant thesaurus terms from Medical Subject Headings (MeSH) and Emtree respectively (see Table 1). In all databases terms were searched in titles and abstracts of references and keywords added by the authors. The search contained terms for (1) tooth extraction or dental/oral surgery and (2) gloves or infection prevention/control. Terms were combined with Boolean operators AND and OR and proximity operators were used to combine terms into phrases. The full search strategies of all databases are available in the Appendix A. The searches in Embase, Medline and Web of Science were limited to exclude conference papers. In all databases (except for Google Scholar) animal only articles were excluded from the search results. No study registries were searched, but Cochrane CENTRAL retrieves the contents of ClinicalTrials.gov and World Health Organization’s International Clinical Trials Registry Platform. No authors or subject experts were contacted, and we did not browse unindexed journals in the field.

The references were imported into EndNote version 21 and duplicates were removed by the medical librarian (EK) using the method as described by Bramer et al. [18]. Consecutively, two reviewers (MMA & JT) independently screened titles and abstracts in EndNote according to the predefined eligibility criteria [19]. Studies judged potentially eligible by either reviewer proceeded to full-text screening, which was also performed independently by both reviewers. Discrepancies at any stage were resolved through discussion until consensus was reached.

**Table 1:** Study characteristics.

Authors (Year)	Study Design	Extraction Type	Participants (Extractions)		Follow-Up	Infection (%)		RR (95% Confidence Interval)
			Sterile	Non-Sterile		Sterile	Non-Sterile	
Acharya et al. (2017) [20]	RCT	Non-surgical	51	51	7 days	0 (0)	0 (0)	NA
Adeyemo et al. (2005) [21]	RCT	Non-surgical	122	147	7 days	0 (0)	5 (3.4)	0.109 (0.006–1.959)
Cheung et al. (2001) [22]	RCT	Non-surgical	271	280	7 days	1 (0.4)	2 (0.7)	0.517 (0.0473–5.664)
Chiu et al. (2005) [23]	RCT	Surgical & non-surgical	137	138	7 days	1 (0.7)	3 (2.2)	0.336 (0.035–3.188)
Deshmukh et al. (2017) [24]	RCT	Non-surgical	50	50	7 days	0 (0)	0 (0)	NA
Giglio et al. (1993) [5]	CCT	Surgical & non-surgical	62 (91)	62 (92)	7 days	0 (0)	0 (0)	NA
Shah et al. (2014) [25]	RCT	Non-surgical	30 (44)	30 (42)	7 days	0 (0)	0 (0)	NA
Totals			672	758		2/672 (0.3%)	10/758 (1.3%)	

NA—non applicable.

### 2.2. Study Selection

For this systematic review, randomized controlled trials, clinically controlled trials and retrospective observational trials that compared sterile to non-sterile glove use in dental extractions were included. Patients in these studies underwent either surgical or non-surgical dental extractions with documentation on glove use and postoperative infected socket. A minimal follow-up of 7 days was required to assess the previously noted complications. Non-surgical extractions were defined as simple forceps extractions without flap elevation, bone removal, or tooth sectioning, while surgical extractions involved any procedure requiring flap reflection, osteotomy, or sectioning of the tooth.

Studies were excluded if they did not directly compare sterile versus non-sterile or clean glove use, did not report postoperative socket infection as an outcome, lacked a minimum follow-up of 7 days, or failed to clearly describe glove type during the extraction procedure. In addition, studies published in languages other than English, inaccessible full-texts after multiple author contact attempts, reviews, case reports, conference papers and animal studies were excluded.

### 2.3. Quality Assessment

All included studies were assessed and reviewed on methodological features by two independent reviewers (MMA & JT) according to the Cochrane Reviewers Handbook. Randomized controlled trials were assessed using the Risk of Bias tool 2 (RoB-2) [26]. This tool guides assessment of randomized controlled trials based on five domains including randomization, intervention deviation, missing outcome data, outcome measurement and reported result selection. For non-randomized trials, the Robins-I tool was used [27]. All assessments were performed by the reviewers independently. In cases of initial disagreement, the reviewers jointly re-examined the full text of the study, discussed the applied judgment criteria, and re-assessed the risk-of-bias rating until consensus was reached. No third reviewer was required.

### 2.4. Data Abstraction and Outcome

The primary outcome of this systematic review was the incidence of post-operative wound infections at 7 days in surgical and non-surgical extractions. From the included studies, data on methodology, patient demographics (smoking, alcohol use, diabetes and immune deficiencies), treatment details (tooth type, operator experience, extraction difficulty and time, use of aseptic measures, antibiotics use, indication for extraction, sterile draping, and instrument sterility) and study outcome were extracted. In addition, we collected information on how post-operative infection was diagnosed in each study, including the clinical criteria used to define an infected socket. Infections were recorded according to the diagnostic criteria defined by each individual study. For the primary outcome, the total number of infections in each study arm was extracted. The total number of patients per study arm was used to calculate the incidence of infections. No data on dry socket or inflammation was collected.

Relative risk (RR) was the unit of analysis for the primary outcome as dichotomous data were compared. Meta-analysis was conducted with Cochrane Review Manager (RevMan) version 5.4, using a random-effects model. A random effects model was used due to the clinical heterogeneity in inclusion criteria across the studies, involving variations such as the inclusion of diabetic patients, patients with smoking and alcohol habits in some studies, and the exclusion of these factors in others. Furthermore, the interventions in the included studies differed, with some including both surgical and non-surgical extractions, while others focused solely on non-surgical extractions. Statistical significance was indicated by *p*-values below 0.05.

## 3. Results

### 3.1. Studies

The initial systematic literature search resulted in 7170 publications which were considered for inclusion. From these, the two reviewers included 52 publications after assessing their titles and abstracts. Among the considered publications, 20 articles were in discordance between the 2 independent reviewers. After deliberation between the reviewers, a consensus was reached to consider 38 articles for full-text review. Publications that were disregarded for full-text review had no clear evidence of providing relevant information on glove use in relation to oral extraction infections.

**Figure 1:**
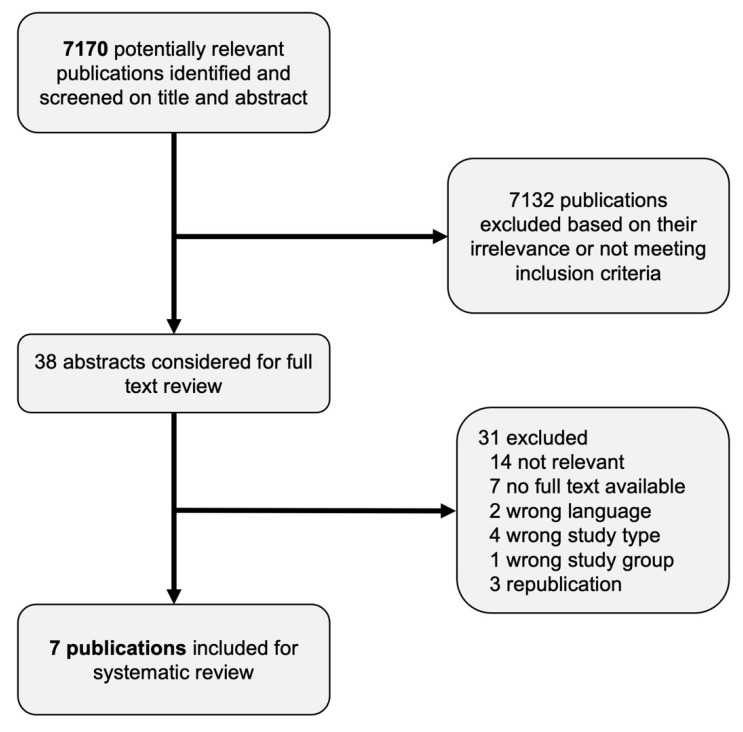
Flow chart of included publications.

Of the remaining 38 publications that underwent full-text review, 7 articles met the inclusion criteria (Figure 1). Multiple attempts were made to contact the authors of publications from which no full-text could be retrieved. As these requests were left unanswered, these articles were excluded as well. There was no discordance between the two independent reviewers in the full-text inclusions.

### 3.2. Patients

The included seven studies encompassed a total of 1430 patients, with participant numbers ranging from 60 to 551. There was clinical heterogeneity in the included patient population. Out of the seven studies, two studies (Cheung et al. and Deshmukh et al.) included patients with smoking and alcohol use habits, of which Cheung et al. also included diabetic patients, including patients with uncontrolled diabetes [22,24]. Among the remaining five studies that excluded patients with smoking or alcohol use, exclusion criteria were inconsistently defined. For example, some explicitly excluded individuals with a history of head and neck radiotherapy or recent systemic antibiotic use, whereas others did not mention these criteria. Similarly, the phrasing used to describe underlying systemic health conditions varied between trials. Adeyemo et al. excluded patients with medical conditions and listed specific disorders, including nutritional deficiencies, endocrine disturbances, and patients using oral contraceptives or steroid therapy [21]. In contrast, Chiu et al. limited exclusions to patients currently on antibiotics for active infection or those requiring antibiotic prophylaxis prior to extraction [23]. These differences demonstrate that baseline patient characteristics and exclusion criteria were interpreted and formulated differently across the included studies.

Two studies (Chiu et al. and Giglio et al.) included both surgical and non-surgical extractions, whereas the remaining five studies investigated only non-surgical extractions [5,23]. Across all included trials, none reported on the use of specific aseptic measures such as pre-operative chlorhexidine, sterile draping, or instrument sterility. Other procedural characteristics—such as indication for tooth extraction, extraction difficulty, operator experience, extraction time, and tooth type—were only reported to a limited degree or not consistently documented. Finally, none of the studies reported the use of postoperative antibiotics, either implicitly or explicitly.

### 3.3. Diagnosis

There was considerable variation in how postoperative infection was defined across the included studies, both in terminology and in the clinical signs used to determine whether a socket was infected. Giglio et al. defined an infected surgical site as the presence of one or more of the following signs: suppuration, erythema, or persistent edema [5]. Adeyemo et al. and Shah et al. used a similar definition: painful socket with suppuration, erythema, and edema with or without systemic fever [21,25]. Cheung et al., in contrast, required systemic fever as part of the diagnostic definition (pain, redness, swelling, pus discharge, and systemic fever), whereas Chiu et al. stated that they applied the criteria described by Cheung et al. but defined infection as redness, swelling, pus discharge or systemic fever, thereby making fever optional rather than mandatory [22,23].

Deshmukh et al. applied a broader and less clearly defined assessment method, listing exudate, odor, peri-socket mucosal appearance, pain characteristics, swelling, fever due to infection, and “presence of an infected socket” but without explicitly specifying which clinical signs constituted a definitive diagnosis [24]. Acharya et al. reported postoperative complications as “dry socket” or “acutely inflamed socket,” but did not provide explicit symptom-based diagnostic criteria for infection [20].

### 3.4. Infection Rate

The infection rates in the sterile glove group ranged from 0% to 3.4% across the included studies. In the sterile group, a total of two infections were reported, resulting in an infection rate of 0.3%. In the non-sterile group, a total of 10 infections were reported, resulting in an infection rate of 1.3%. Regarding the infection rates in surgical and non-surgical extractions, only Chiu et al. and Giglio et al. included both extraction types [5,23].

**Figure 2:**
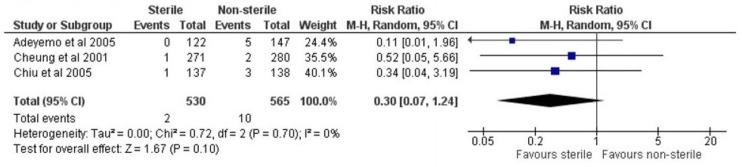
Forest plot [21,22,23].

In total, three studies were eligible for meta-analysis. Other studies could not be considered as they reported no events in either the intervention or control group which impeded further statistical analysis. Such double-zero studies do not contribute information on the direction or magnitude of treatment effect and therefore cannot contribute to the pooled relative risk estimate. A total of 1068 patients were included in the meta-analysis which resulted in a pooled relative risk ratio of 0.30 (95% CI 0.07–1.24) (Figure 2). No statistical heterogeneity was detected among the included studies, with an I2 of 0%. In addition, among the studies that could be included in the meta-analysis, only Chiu et al. contained both surgical and non-surgical extractions, so no distinction between surgical and non-surgical procedures could be made in the pooled analysis.

### 3.5. Risk of Bias

There were minor discrepancies in the quality assessment between the two reviewers in certain instances. Nevertheless, through deliberation, a consensus was reached without the need for involvement from a third reviewer. All studies were collectively categorized as having moderate to low quality, assessed through the RoB-2 or ROBINS-1 tool (Table 2 and Table 3).

## 4. Discussion

The use of sterile gloves in surgical and non-surgical dental extractions has been a long-standing topic of interest, with both clinical and environmental implications. In this systematic review and meta-analysis, we aimed to assess the impact of sterile versus non-sterile gloves on post-operative infections following dental extractions. Ultimately, we found no difference in post-operative infection rates between dental extractions performed with sterile gloves compared to non-sterile gloves. Our findings contribute to the ongoing debate surrounding glove usage and its potential implications for patient safety and infection prevention.

A general concern when evaluating the use of sterile gloves in dental extractions is the prevention of post-operative wound infections. In our analysis of the included studies, we observed a relatively low overall infection rate in both the sterile and non-sterile glove groups. Upon comparing these rates, no statistically significant difference was found. Notably, the infection rates across studies varied, with some studies reporting no infections in either group. Studies in which both groups reported zero infections could not be included in the meta-analysis because such trials do not contribute information on the direction or magnitude of a relative effect, and performing continuity corrections would not meaningfully improve interpretability. Moreover, both the included and excluded studies comprised small sample sizes and were therefore likely underpowered to detect any post-operative infection. Even the studies that did report infections are likely to be underpowered, as substantially larger sample sizes would be required to detect a statistically meaningful difference between incidence rates as low as 0.3% and 1.3%. As a result, the absence of a statistically significant difference should be interpreted cautiously, as it may reflect limited statistical power rather than indicating a true lack of difference between sterile and non-sterile glove use. In this context, the finding of an I^2^ value of 0% should also be interpreted with care. With only three studies contributing data and low event rates, the statistical power to detect between-study heterogeneity is extremely limited. An I^2^ of 0% therefore reflects an absence of detectable heterogeneity rather than true clinical homogeneity.

The fact that this study found low infection rates in both groups could hint that regardless of any statistically significant difference, sterile gloves should not be used based on the numbers needed to treat. If we hypothesize that there is a difference between infection rates after dental extractions performed with either sterile or non-sterile gloves, we calculated that this would result in an approximate number needed to treat of 100 cases. As treatment usually involves a dentist or oral surgeon and an assistant, 200 pairs of sterile gloves would be required to prevent one patient from developing a post-operative socket infection. In general, socket infections are relatively mild and can be treated surgically or conservatively with or without the use of antibiotics. Therefore, regardless of any significant statistical difference, it is debatable whether there is a clinical ground to prefer sterile gloves over non-sterile gloves in dental extractions.

An important point of consideration is the absence of data regarding the differences in infection rates between surgical and non-surgical extractions. In the current study only Chiu et al. and Giglio et al. included both extraction types but neither could provide any subdivision in their infection rates, possibly due to the low event rate. Moreover, among the studies that could be included in the meta-analysis, only one contained surgical extractions. Although a subgroup analysis for surgical versus non-surgical extractions was considered, this was not statistically feasible due to the limited number of surgical cases and the absence of stratified outcome reporting. Therefore, we must conclude that our meta-analysis does not offer sufficient data to distinguish between the two extraction types. This distinction is clinically relevant. Surgical extractions inherently cause more tissue damage and disruption of the natural mechanical barrier compared to non-surgical extractions. As a result of this disruption, tissues could become more vulnerable to bacterial and pathogenic exposure, potentially leading to post-procedural infections. Therefore, aseptic measures could be more relevant by limiting the number of bacteria distributed by health care professionals. This hypothesis seems to be supported by previous research that showed surgical and complex extractions to be associated with higher infection rates [28]. However, the low overall incidence of postoperative infections reported across the included studies limits the ability to determine whether sterile gloves would have a significant effect in surgical versus non-surgical extractions. Consequently, it remains unclear whether the use of sterile gloves meaningfully reduces the risk of postoperative infection in either extraction type.

From a microbiological view, the rationale for the use of sterile gloves and the subsequent sterile setup in dental extractions is debatable. As previously noted, the oral cavity houses over 700 aerobic and anaerobic types of bacteria. During dental procedures, gloves are in direct contact with oral mucosa and saliva, causing subsequent contamination. The susceptibility to microbial contamination is similar for sterile and non-sterile gloves upon entering the oral cavity. Therefore, it is unlikely that a sterile glove can significantly reduce the microbial film in extraction sockets to limit post-operative infections. In addition, evidence demonstrates that there is no significant difference in bacterial contamination, either bacteria counts or type, or post-operative infection rates between sterile and non-sterile gloves in dental extractions and in minor outpatient surgery [5,24]. Furthermore, a previous meta-analysis, including over 11,000 patients, on the use of sterile gloves in outpatient skin surgery showed no advantage in infectious complications over non-sterile gloves. Given that the oral cavity harbors a higher bacterial load than intact skin and yet no additional benefit of sterile gloves was observed in skin surgery, it is reasonable to assume that a similar lack of added benefit may apply to dental extractions, which are performed in an inherently contaminated field, for both surgical and non-surgical procedures [29,30,31].

Alternatively, it could be asserted that wearing sterile gloves is essential to inhibit the introduction of new bacteria into the mouth, thereby preventing nosocomial infections. Nevertheless, in surgical or non-surgical extractions, a direct link is established between the dental socket and the surrounding microbiome. In this respect, the resulting wounds are comparable. Considering that non-sterile gloves are successfully used in uncomplicated dental extractions, one can expect comparable outcomes in surgical extractions. Moreover, given there was no evidence to suggest that glove cross-contamination significantly affected post-operative infection rates in non-surgical extractions, it is plausible that a similar limited impact on infections would be observed in surgical settings.

Healthcare systems are wasteful and contribute significantly to environmental pollution. In light of sustainability efforts and reducing current waste trends, current practices involving a high turnover of disposable plastics should be critically reconsidered. Dental procedures are of specific importance as these are one of the most commonly performed outpatient procedures worldwide. Sterile gloves are routinely used within dental care, especially in surgical procedures. Compared to non-sterile gloves, these sterile gloves generate 11.6-fold more pollution and are more expensive [10]. This study contributes clinical context to ongoing discussions on sustainability in healthcare and may support further research specifically aimed at quantifying the environmental impact of disposable materials in dental practice.

This review was subject to a couple of limitations due to the underlying articles included. First, the included studies were of relatively low to moderate quality which limits the generalizability of this review. Especially in studies in which observers of primary outcomes were not blinded for treatment given, we concluded that risk of bias was high. This lack of blinding may have influenced the subjective assessment of postoperative infection signs such as pain, swelling, or erythema, potentially leading to differential outcome assessment between study groups and over- or underestimation of true infection rates. In addition, the heterogeneity in diagnostic criteria used to define postoperative infection across studies further limits the comparability of outcomes and may have resulted in outcome misclassification, thereby increasing uncertainty regarding the accuracy and comparability of the found infection rates.

Second, the included studies were subject to confounding as some included diabetic patients or patients with smoking habits, and the indication for dental extraction was not mentioned. These patient-related factors are independently associated with impaired wound healing and may therefore have acted as inadequately measured effect modifiers [32,33]. The inability to control for these confounders prevents isolation of the independent effect of glove sterility on postoperative infection risk and introduces uncertainty into the direction and magnitude of the observed associations.

Third, several key procedural and perioperative variables were either not reported or reported inconsistently across the included studies. None of the studies specified whether chlorhexidine mouthwash was used pre-operatively, whether sterile draping was applied, or whether sterile instruments were used. In addition, other relevant procedural factors—including tooth type, indication for extraction, extraction difficulty, procedure duration, and operator experience—were poorly documented or entirely absent. Because each of these factors may independently influence bacterial exposure and postoperative infection risk, their incomplete reporting limits comparison between studies and precludes adequate adjustment for procedural complexity. Consequently, any observed effect, or lack thereof, cannot be confidently attributed to glove sterility alone.

Fourth, subject experts were not contacted and hand-searching was not performed in the search strategy, which may have resulted in the omission of a limited number of relevant unpublished or non-indexed studies. However, given the extensive electronic database search strategy and the large yield of 7170 records identified, the likelihood that these supplementary methods would have resulted in the identification of a substantial number of additional eligible studies is considered limited unlikely to affect the conclusions.

Additionally, there is a need for further research to assess surgeons’ preferred gloves, as this aspect was not sufficiently addressed in the current literature. This is a relevant aspect, as user experience could play a significant role in decision making on plastic use in healthcare.

Finally, as previously mentioned, the included studies were underpowered and even combined since insufficient patient numbers were available to demonstrate statistically significant results given the low incidence of infections. To reduce potential bias and the presentation of skewed data, a multi-center randomized clinical trial with large numbers is required. As this review illustrated, reaching these numbers can be challenging from a financial as well as from a logistic perspective. However, as more literature is still needed to assist in decision making on glove use within (surgical) dental extractions, one could opt for second-best alternatives in research. An alternative research method could include comparing two historical cohorts in hospitals that transitioned from using sterile gloves to non-sterile gloves in (surgical) dental extractions. If such a research method could be set up in a multi-center fashion, valuable data can be obtained without the challenges of a large multi-center randomized clinical trial.

In conclusion, within the various limitations of this study, including low event rates, small sample sizes, heterogeneity in diagnostic definitions, and the absence of outcome data on surgical extractions outcome, the current evidence does not demonstrate a difference in postoperative infection rates between sterile and non-sterile gloves in dental extractions. We recommend that future research include large, adequately powered studies—preferably multi-center trials—that separately evaluate surgical and non-surgical extractions and apply standardized diagnostic criteria for postoperative infection.

## Figures and Tables

**Table 2:** Quality assessment—RoB 2 tool.

Authors (Year)	Randomization Process	Deviations from Intended Intervention	Missing Outcome Data	Measurement of Outcome	Selective Reporting	Overall Bias
Acharya et al. (2017) [20]	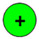	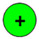	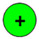	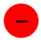	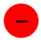	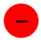
Adeyemo et al. (2005) [21]	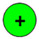	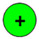	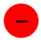	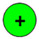	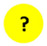	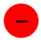
Cheung et al. (2001) [22]	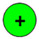	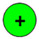	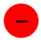	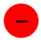	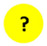	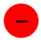
Chiu et al. (2005) [23]	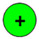	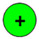	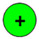	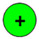	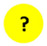	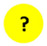
Deshmukh (2017) [24]	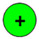	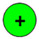	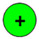	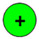	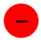	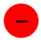
Shah (2014) [25]	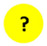	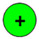	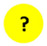	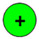	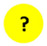	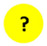

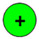
: Low risk. 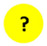: Some concerns. 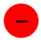: High risk.

**Table 3:** Quality assessment—ROBINS-1 tool.

Authors (Year)	Bias Due to Confounding	Bias in Selection of Participants into the Study	Bias in Classification of Interventions	Bias Due to Deviations from Intended Intervention	Bias Due to Missing Data	Bias in Measurement of Outcomes	Bias in Selection of Reported Result	Overall Bias
Giglio et al. (2017) [5]	Serious	Low	Low	Low	Low	Moderate	Moderate	Moderate

## Data Availability

No new data were created or analyzed in this study.

## Appendix A

Embase	(‘tooth extraction’/exp OR ‘dental surgery’/de OR ‘oral surgery’/de OR (exodontia* OR exodontic* OR odontectom* OR ((tooth* OR teeth OR dental* OR molar* OR oral* OR maxillofacial*) NEAR/3 (extract* OR amputat* OR remov* OR resect* OR surg*))):ab,ti,kw) AND (‘glove’/exp OR ‘infection prevention’/exp ‘infection control’/exp OR (glov* OR steril* OR antisepsis* OR asepsis* OR ((infection* OR infectious* OR infected* OR postinfect* OR complicat*) NEAR/6 (prevent* OR prophylax* OR reduc* OR decreas* OR control* OR increas*))):ab,ti,kw) NOT ((animal/exp OR animal*:de OR nonhuman/de) NOT (‘human’/exp)) NOT ([Conference Abstract]/lim OR [preprint]/lim)
Medline	(exp tooth extraction/OR Surgery, Oral/OR (exodontia* OR exodontic* OR odontectom* OR ((tooth* OR teeth OR dental* OR molar* OR oral* OR maxillofacial*) ADJ3 (extract* OR amputat* OR remov* OR resect* OR surg*))).ab,ti,kf.) AND (exp Gloves, Protective/OR exp Infection Control/OR (glov* OR steril* OR antisepsis* OR asepsis* OR ((infection* OR infectious* OR infected* OR postinfect* OR complicat*) ADJ6 (prevent* OR prophylax* OR reduc* OR decreas* OR control* OR increas*))).ab,ti,kf.) NOT (exp animals/ NOT humans/) NOT (news OR congres* OR abstract* OR book* OR chapter* OR dissertation abstract*).pt.
Cochrane	((exodontia* OR exodontic* OR odontectom* OR ((tooth* OR teeth OR dental* OR molar* OR oral* OR maxillofacial*) NEAR/3 (extract* OR amputat* OR remov* OR resect* OR surg*))):ab,ti,kw) AND ((glov* OR steril* OR antisepsis* OR asepsis* OR ((infection* OR infectious* OR infected* OR postinfect* OR complicat*) NEAR/6 (prevent* OR prophylax* OR reduc* OR decreas* OR control* OR increas*))):ab,ti,kw)
Web of Science	TS = (((exodontia* OR exodontic* OR odontectom* OR ((tooth* OR teeth OR dental* OR molar* OR oral* OR maxillofacial*) NEAR/2 (extract* OR amputat* OR remov* OR resect* OR surg*)))) AND ((glov* OR steril* OR antisepsis* OR asepsis* OR ((infection* OR infectious* OR infected* OR postinfect* OR complicat*) NEAR/5 (prevent* OR prophylax* OR reduc* OR decreas* OR control* OR increas*)))) NOT ((animal* OR rat OR rats OR mouse OR mice OR murine OR dog OR dogs OR canine OR cat OR cats OR feline OR rabbit OR cow OR cows OR bovine OR rodent* OR sheep OR ovine OR pig OR swine OR porcine OR veterinar* OR chick* OR zebrafish* OR baboon* OR nonhuman* OR primate* OR cattle* OR goose OR geese OR duck OR macaque* OR avian* OR bird* OR fish*) NOT (human* OR patient* OR women OR woman OR men OR man))) NOT DT = (Meeting Abstract OR Meeting Summary)
Google Scholar	exodontia|exodontics|odontectomy|”tooth|teeth|dental|molar|oral|maxillofacial extraction|amputatation|removal|resection|surgery” glove|gloves
